# Is there a delay in seeking medical care after the first seizure in “resource limited settings”: a pilot study from Sri Lanka

**DOI:** 10.1186/s13104-018-3887-3

**Published:** 2018-10-29

**Authors:** H. M. M. T. B. Herath, Milinda Withana, Ranjani Gamage, Chaturaka Rodrigo

**Affiliations:** 1 Institute of Neurology, National Hospital of Sri Lanka, Colombo 10, Sri Lanka; 2 Department of Clinical Medicine, Faculty of Medicine, University of Colombo, Colombo, Sri Lanka; 3 Department of Pathology, School of Medical Sciences, UNSW, Sydney, Australia

**Keywords:** Epilepsy, Treatment delay, Sri Lanka, Diagnosis, First seizure

## Abstract

**Objectives:**

Current guidelines suggest that patients presenting with the first seizure should be assessed by a specialist, preferably with investigations such as electroencephalography and imaging to reach a definitive diagnosis. We conducted a cross sectional study among patients with confirmed epilepsy, at a tertiary level neurology clinic in Sri Lanka with the aim of assessing delays in first contact with a medical doctor and in performing key investigations after the first seizure.

**Results:**

Majority had sought medical attention within 24 h of the first seizure (71.2%) and had seen a specialist within the 1st week since the seizure (61%). Also a significant proportion had completed key investigations such as electroencephalography (63.2%) and brain imaging within a month (51%) since the first medical consultation. Of many socio-demographic and illness related factors examined, only a non-generalized tonic–clonic presentation was significantly associated with delay in seeking medical help.

## Introduction

Current guidelines suggest that patients presenting with first seizure should be assessed by a specialist as soon as possible, preferably with an electroencephalogram (EEG) and imaging to reach a definitive diagnosis [1]. However, the geographical location, cultural beliefs, available health care facilities and individual health seeking behaviours of patients can either facilitate or restrict this process [2, 3].

The delays in receiving appropriate medical care can be due to patient related or healthcare provider related factors. On patient related factors, stigma and unawareness associated with epilepsy plays a major role in denial of diagnosis and seeking alternative treatment [4, 5]. Regarding healthcare provider related factors, lack of qualified medical practitioners and specialists, reduced accessibility to specialist care as well as unavailability or high demand for investigations can lead to delays. The first step in addressing any shortcomings in treatment delays would be to quantify the extent of the problem. We conducted a cross sectional study among patients with confirmed epilepsy who are followed up at a tertiary level neurology clinic in Sri Lanka with the aim of assessing delays in first contact with a medical doctor and in completing key investigations (EEG and MRI) after the first seizure.

## Main text

This cross-sectional survey was conducted at the National Hospital of Sri Lanka (NHSL) from April to July 2016. The NHSL is the largest tertiary care referral centre in Sri Lanka. The institute of Neurology of this hospital has two general neurology units which collectively receives referrals from all over the country and this study included one of them (unit 1). It also serves the most populous Colombo district of Sri Lanka which consists of 11.4% of the total population in Sri Lanka [6], with regard to general neurological patient care. Epilepsy patients are usually followed up at clinics held twice a week and all these clinics were covered in the study. Each patient however, attends his/her clinic at a monthly frequency to get a month’s supply of drugs. Hence the study duration of 3 months was considered adequate as all eligible patients including anyone who missed 1 or 2 consecutive clinic visits were likely to be interviewed. Both neurology units are on-call every other day and receive all in-patient and out-patient referrals while on-call without any specific preferences for a particular type of a neurological disorder. All patients referred to a unit initially are followed up in that unit’s clinic. Hence the patient cohort profile of the unit that was studied is expected to be similar to the other unit which was not assessed.

This study included all consenting adult (over 12 years of age) patients with a diagnosis of epilepsy confirmed by EEG and currently on anti-epileptic medication, followed up at clinics of neurology unit 1 of NHSL. All patients meeting the inclusion criteria were invited to participate. Exclusion criteria were undiagnosed seizures, clinically diagnosed epilepsy without EEG evidence and non consenting patients. The data collection instrument was an interviewer administered, pre tested questionnaire that collected data on basic demographic variables (age, sex, occupation, income), illness related factors (diagnosis, duration of illness, medication and investigations), and delays in seeking treatment as well as perceived reasons for this delay.

The data analysis was carried out with SPSS statistical software (Version 23, IBM, USA), and significance of associations were calculated using Chi square test/Fishers exact test and independent T test. A p value < 0.05 was considered statistically significant.

One hundred and forty-two patients met the inclusion criteria and 118 patients participated in the survey with an 83% response rate. The participants had an equal gender distribution (males: 60/118, 50.8%) with a mean age of 32.8 years (SD ± 14.3). The demographic characteristics of the sample are summarized in Table 1. Overall, a majority were employed and had received at least 11 years of formal school education.

**Table 1:** Demographic characteristics of the participants (n-118)

Characteristic	Number	Percentage (%)
Gender		
Male	60	50.8
Female	58	49.2
Civil status		
Married	54	45.8
Single	62	52.5
Divorced	2	1.7
Employment		
Currently working	60	50.8
Student	15	12.7
Retired/not working	43	36.4
Residence		
Within Colombo district	61	51.7
Outside Colombo district	57	48.3
Per capita income per month (LKR)		
Less than 10,750	16	13.6
More than 10,750	102	86.4
Level of education (n-100)		
Primary or no formal education	17	17.0
Secondary education	71	71.0
Tertiary education	12	12.0

Regarding the seizure type, most had complex partial seizures (40, 33.9%), followed by generalized tonic–clonic seizures (39, 33.1%) and secondary generalized seizures (31, 26.3%). Interestingly, 84 (71.2%) patients had sought medical attention within the following 24 h since the first seizure (Table 2). In fact, a majority (62, 52.5%) of these consultations were with a consultant neurologist and by 1 month, a total of 86 (74.1%) patients were seen by a consultant neurologist. However, only a small proportion of patients (28, 24.6%) had undergone an EEG in the 1st week since the medical consultation. Yet, a majority (88, 77.2%) has had at least one EEG by 3 months. Despite the limited availability of MRI facilities, 55 patients (46.7%) had received their first magnetic resonance imaging (MRI) scan within 3 months of the first seizure which increased to 70 (59.3%) at 6 months (Table 2). Eighty patients (67.6%) had contacted a medical practitioner after the first seizure and a further eleven patients reported to medical care after a second seizure (total: 91, 77.1%). However, a small proportion (17, 14.4%) did not contact a doctor until they had five or more separate seizures. When probed about the reasons for delay, seizure unawareness was the most commonly cited reason (28, 77.7%) by those who waited for more than one seizure to contact a doctor. Other responses included; being busy (4, 4.5%), potential negative social impact of an epilepsy diagnosis (2, 2.8%) and trying traditional/alternative treatment first (2.2.8%).

**Table 2:** Illness related characteristics and delays in seeking treatment (n-118)

Characteristic	Number	Percentage (%)
Seizure phenotype		
Generalized tonic–clonic	39	33.1
Partial (complex)	40	33.9
Partial (simple)	4	3.4
Secondary generalization	31	26.3
Other	4	3.4
Time lapse till medical attention after the first seizure		
Within 24 h	84	71.2
Within 1 week	10	8.5
One week to 1 month	10	8.5
One month to 1 year	6	5.1
More than a year	8	6.8
Time lapse still first specialist consultation after the first seizure (n-116)		
Within 24 h	62	53.4
Within 1 week	9	7.6
One week to 1 month	15	12.9
One month to 1 year	12	10.4
More than a year	18	15.6
Time lapse from first medical consultation to first EEG (n-108)		
Within 1 week	28	24.6
One week to 1 month	44	38.6
One month to 1 year	25	21.9
More than a year	10	8.8
Time lapse from first medical consultation to first MRI (n-96)		
Within 1 week	16	14.8
Within 1 month	39	36.1
Within 1 year	25	23.2
More than a year	16	14.8

There was no significant association between delay in seeking medical help (> 1 week lapse to first medical consultation since the first seizure) and any of the following factors; marital status, monthly income below mean average personal income [7], employment, no schooling or completing primary education only and being an adult (> 12 years of age) at the onset of seizures. However having a non-generalized tonic–clonic seizure phenotype was associated with a delay in consulting a doctor (p = 0.03).

In summary, more than 70% of patients sought medical attention within 24 h of the first seizure and 74% were seen by a consultant neurologist within a month of the event. Regarding investigations, 77% had their first EEG completed within 3 months and 60% had at least one MRI scan within 6 months of the first medical consultation.

Early consultation with an epileptologist after the first seizure and a comprehensive follow up with investigations such as EEG and MRI scanning has shown benefit for a faster diagnosis, better patient compliance with anti-epileptic medication and to delay a recurrence of seizures (for up to 12 months of follow up) [8]. However, this ideal picture is difficult to achieve in the “real world”. In a survey in Australia, 220 patients presenting with seizures were investigated for previous events that was either a seizure or a non-convulsive manifestation of a seizure. Of this sample from an urban community (in Melbourne), approximately 36% presented at least 4 weeks after the first event and 14% presented 2 weeks later. Non convulsive seizures and socioeconomic disadvantage were associated with a delayed presentation [9]. In contrast, a study in India showed that more than 90% of patients were seen by a medical practitioner hours after the seizure [10]. While generalizations cannot be made by small studies, it is interesting that in our study as well, a majority of patients received medical attention within 24 h of the first seizure and even more so, a majority was seen by a specialist neurologist within a month. We believe that this result is very interesting as it highlights the accessibility to specialists in a country where 21 million people are served by approximately 35 practicing neurologists (including paediatric neurologists) in government hospitals [11]. Even if retired neurologists who are still practicing are counted, the number is unlikely to exceed 50. This is in contrast to Sydney (142 neurologists per 4.3 million people in 2009) or Melbourne (123 neurologists per 3.8 million people in 2009) in Australia which has a much favourable neurologist per population ratio [12]. Currently, there are no epileptologists in Sri Lanka.

The reason behind accessibility is probably the “hybrid” healthcare delivery system in Sri Lanka where people can choose whether to receive care in the government (free of charge) or in the private sector. The government sector has a range of healthcare institutions varying from primary health care units, divisional hospitals, base hospitals, district general hospitals (n-19), provincial general hospitals (n-3) and teaching hospitals (n-19) [13]. Neurology services are currently available in some teaching hospitals, provincial general hospitals and some district general hospitals only. Consultant general physicians, who are available from base hospitals upwards, fill in for the neurology services in the periphery. To access a neurologist from one of the peripheral stations, a “bottom to top” referral system by other medical practitioners is a must. However, this is not the case in the private healthcare sector. The same specialists who serve in government hospitals are accessible in the private sector after regular working hours (8 a.m.–4 p.m.) and the patient can decide whom he or she wants to consult. A consultation fee can vary from 10 to 20 USD which is affordable to most citizens provided visits are done at a frequency of once a month or less [7]. Some patients may get referred to the public health sector to continue the medication after seen by a specialist in the private sector. Though this system is not ideal and leads to overcrowding in private sector without a proper triaging system, it benefits patients in terms of accessibility. Currently the estimated prevalence of epilepsy in Sri Lanka is 0.3% (approximately 63,000 patients) [14] and it is impossible to handle such a large volume of patients in the government sector alone. The evolution and survival of this unique healthcare delivery system, which has shaped itself by need rather than design, seems to be fulfilling the needs of the community in a resource limited setting.

In conclusion, despite being a densely populated country of 21 million people having approximately 1 neurologist for 600,000 people [15] and only a handful of MRI scanners, the findings of this study are impressive with regard to the health care seeking behaviour and healthcare delivery after the first seizure. These findings also suggest that the public and private healthcare delivery systems in Sri Lanka are complimenting each other.

## Limitations

This study was conducted in one centre in Sri Lanka and is not representative of all epilepsy patients in Sri Lanka. The situation may vary in different regions in Sri Lanka according to available facilities and cultural beliefs. This study is biased to patients already in the hospital system and there may be large number of epileptics in the community who have not yet consulted a medical officer (or not having regular follow up despite seeing one) and these findings are not representative of that group.

## Abbreviations

EEG: electroencephalography
MRI: magnetic resonance imaging
SD: standard deviation
